# Exploring the Role of Indirect Coupling in Complex Networks: The Emergence of Chaos and Entropy in Fractional Discrete Nodes

**DOI:** 10.3390/e25060866

**Published:** 2023-05-29

**Authors:** Ernesto Zambrano-Serrano, Miguel Angel Platas-Garza, Cornelio Posadas-Castillo, Adrian Arellano-Delgado, César Cruz-Hernández

**Affiliations:** 1 Facultad de Ingeniería Mecánica y Eléctrica, Universidad Autónoma de Nuevo León, San Nicolás de los Garza 66455, NL, Mexico; ernesto.zambranos@uanl.edu.mx (E.Z.-S.); miguel.platasgrz@uanl.edu.mx (M.A.P.-G.); 2 National Council of Science and Technology, Ciudad de Mexico 03940, Mexico; 3 Engineering, Architecture and Design Faculty, Autonomous University of Baja California, Ensenada 22860, BC, Mexico; 4 Electronics and Telecommunication Department, Scientific Research and Advanced Studies Center of Ensenada, Ensenada 22860, BC, Mexico

**Keywords:** complex systems, Caputo-like difference operator, fractional calculus, indirect coupling, chaos, spectral entropy, FPGA

## Abstract

Understanding the dynamics of complex systems defined in the sense of Caputo, such as fractional differences, is crucial for predicting their behavior and improving their functionality. In this paper, the emergence of chaos in complex dynamical networks with indirect coupling and discrete systems, both utilizing fractional order, is presented. The study employs indirect coupling to produce complex dynamics in the network, where the connection between the nodes occurs through intermediate fractional order nodes. The temporal series, phase planes, bifurcation diagrams, and Lyapunov exponent are considered to analyze the inherent dynamics of the network. Analyzing the spectral entropy of the chaotic series generated, the complexity of the network is quantified. As a final step, we demonstrate the feasibility of implementing the complex network. It is implemented on a field-programmable gate array (FPGA), which confirms its hardware realizability.

## 1. Introduction

The exchange of information has been a fundamental aspect of communication, and there has always been a tendency to find more efficient ways of exchanging information. This goal has led to the development of various technologies and systems that enable communication among individuals, as well as between animals, objects, and other entities.

In graph theory, the components of a system that exchange information are called nodes, and the medium of exchange is known as link or coupling. These concepts apply to any network with two or more nodes, such as social networks [1,2], transportation networks [3,4], biological networks [5,6], and neural networks [7]. In a complex network, nodes can be directly coupled with each other, or they can be linked through intermediary systems [8]. These intermediary systems are dynamic systems that decouple the direct interaction between two or more nodes, and they can be chosen based on the specific application to be implemented. Examples of intermediary systems include routers in computer networks and servers in wireless communication networks. Couplings with intermediary systems have been shown to perform better than direct couplings in some cases, as demonstrated in studies such as [9,10]. One important measure of network performance is synchronization, which refers to the tendency of nodes in a network to behave in a coordinated manner. Improving synchronization is particularly important in networks where nodes are chaotic, as it can help stabilize the system and improve its overall performance [11,12,13]. Chaotic systems are characterized by their sensitivity to initial conditions, meaning that a small change in the initial conditions of the system can result in a significantly different output [14]. The study in [15] deals with the coupling of hyperchaotic systems through an intermediary dynamic system, in [8] the coupling of small-world networks through intermediary dynamic systems is presented, and in [16] the enhancing of hyperchaotic dynamics generated by coupling two discrete periodic systems is addressed.

Fractional calculus has become an increasingly important field of study due to its application in various fields, such as mathematics, physics, engineering, and biology [17,18,19]. It is a generalization of traditional calculus that extends the concept of derivatives and integrals to non-integer orders. It has been demonstrated to be a powerful tool in analyzing complex systems, particularly when they exhibit memory and long-range dependence [20]. For instance, the fractional order in an N-type blood vessel model in [21] reveals rich dynamics and faster adaptive synchronization than its integer-order model. The synchronization in multiplex neuronal networks integrated with fractional order Hindmarsh–Rose neurons synchronizes better than integer-order models [22]. In [23], dynamic coupling for fractional order systems is presented. Although chaos and synchronization have been analyzed in complex continuous-time networks, their existence and features in discrete-time systems have also been a subject of interest. Discrete chaotic systems are particularly interesting due to their ability to generate complex behavior from simple rules, making them ideal candidates for use as nodes in complex networks [24,25]. By constructing networks composed of such systems, it is possible to investigate the collective behavior of the network and explore the emergence of complex phenomena [26].

Since the seminal paper of Miller and Ross [27], the idea of discrete fractional calculus and the theory of fractional differences equations has caught the attention of scholars. Similar to fractional order integration and differentiation operators in continuous time, there are different definitions related to discrete fractional calculus, such as the Riemann–Liouville-like difference [28], the Grünwald–Letnikov-like difference [29], and the *q*-difference operator [30]. The Caputo-like delta difference operator is a specific type of discrete fractional calculus that considers the function’s initial values and its delta derivatives. Several studies have shown that the Caputo-like delta difference operator has better numerical stability and accuracy than other fractional difference operators, making it a useful tool for solving fractional difference equations in various applications. A fractional predator–prey discrete system of the Leslie type considering the Caputo-like delta difference operator is proposed in [31]. Ref. [32] introduces a COVID-19 model that incorporates the number of immunized individuals as an additional state variable describing the system dynamics. In [33], the outer synchronization problem of discrete fractional complex networks with and without unknown topology is established based on linear matrix inequalities. Considering the Caputo-like delta difference definition, a fractional difference order map with chaotic dynamics and with no equilibria is proposed in [34].

However, there are not enough studies describing the relationship between the fractional order and the dynamics of the complex network with discrete nodes. As a result, the emergence of chaos in complex dynamic networks with fractional-order discrete systems, where the connection between the nodes is not direct but occurs through an intermediate node, has been considered. Then we provide the fractional order version of the complex network with indirect coupling based on the Caputo-like delta difference operator. The dynamics behaviors associated with the fractional order difference system are analyzed by employing the temporal series, phase portraits, bifurcation diagrams, and the largest Lyapunov exponent, where the numerical simulations illustrate the results of our analysis. Moreover, we analyze the spectral entropy of the network, which quantifies the amount of information generated by the system over time to confirm the rich dynamics of both complex network and indirect coupling in the fractional sense. Finally, we implement the system on a field-programmable gate array (FPGA), which provides a hardware-based system implementation. This allows us to study the behavior of the network to experimentally reproduce their dynamics, exploiting their chaotic properties in real phenomena and providing a platform for further optimization and control. The rest of the paper is organized as follows. Section 2 provides a brief review of the Caputo derivative in discrete-time systems. Section 3 presents the methodology to generalize the complex network. Section 4 gives the dynamic analysis of the fractional order network with indirect coupling. Section 5 provides the complexity analysis via spectral entropy. In Section 6, we implement the system on a field-programmable gate array. Finally, Section 7 provides concluding remarks and future research directions.

## 2. Preliminaries

This section presents definitions, theorems, and remarks for use in the paper. Subsequently, we will consider the general *n*-th order difference, which can be expressed as

(1) $$\begin{array}{cc} \Delta^{n}f\left(t\right) & =\Delta^{n-1}f\left(t+1\right)-\Delta^{n-1}f\left(t\right),\\ \; & =\sum_{k=0}^{n}C_{n}^{k}{\left(-1\right)}^{k}f\left(t+n-k\right), \end{array}$$

where $C_{n}^{k}$ is the binomial coefficient, $C_{n}^{k}=\frac{k!}{n!(n-k)!}$. Expanding the concept to fractional order difference, the fractional sum of order *v* is described as follows.

**Definition** **1** ([35]). *If f(t) is a real-valued function defined on the discrete set $N_{\phi }=\left\{\phi ,\phi +1,\phi +2,\ldots \right\}$ with ϕ∈R and v>0, then, the v-fractional order sum of f(t) denoted as $\Delta_{\phi }^{-v}$ is defined as*

(2) $$\Delta_{\phi }^{-v}f\left(t\right)=\frac{1}{\Gamma (v)}\sum_{s=\phi }^{t-v}{\left(t-\sigma \left(s\right)\right)}^{v-1}f\left(s\right),\;t\in N_{\phi +v},$$

*where ϕ is the starting point, the forward operator $\phi :N_{\phi }\to N_{\phi }$ given as σ(s)=s+1, $t^{\left(v\right)}=\frac{\Gamma (t+1)}{\Gamma (t+1-v)},$ with t≠−1,−2,−3,…, is the falling function, and Γ(·) is the gamma function, denoted as $\Gamma \left(z\right)=\int_{0}^{\infty }e^{-t}t^{z-1}dt$. Below, we will consider the Caputo derivative in vector form as the fractional difference.*

**Definition** **2** ([36]). *Let v>0 with v∈N. The v-order Caputo-like discrete fractional difference of a function f(t) defined on $N_{\phi }$ is denoted by*

(3) $$\begin{array}{ll} {}^{C}\Delta_{\phi }^{v}f\left(t\right) & =\Delta_{\phi }^{-(m-v)}\Delta^{m}f\left(t\right),\\ & =\frac{1}{\Gamma (m-v)}\sum_{s=\phi }^{\left(t-(m-v)\right)}{\left(t-\sigma \left(s\right)\right)}^{\left(m-v-1\right)}\Delta^{m}f\left(s\right), \end{array}$$

*where $t\in N_{\phi +m-v}$, with $m=\left\lceil v\right\rceil +1$, v the fractional order, and ϕ the lower bound.*

The *v*-th Caputo-like delta discrete fractional difference is defined specifically for the case when m=1, as follows:

(4) $${}^{C}\Delta_{\phi }^{v}f\left(t\right)=\frac{1}{\Gamma (1-v)}\sum_{s=\phi }^{\left(t-(1-v)\right)}{\left(t-\sigma \left(s\right)\right)}^{\left(-v\right)}\Delta f\left(s\right),$$

with $t\in N_{\phi +1-v}.$

**Theorem** **1** ([37]). *For the nonlinear fractional Caputo-like difference equation*

(5) $$\begin{array}{ll} {}^{C}\Delta_{\phi }^{v}f\left(t\right) & =f(t+v-1,u(t+v-1)),\\ \Delta^{k}u\left(\phi \right) & =u_{k}, \end{array}$$

*with $m=\left\lceil v\right\rceil +1,$k=0,…,m−1. For $t\in N_{\phi +m}$, the discrete integral equation equivalent can be represented as*

(6) $$\begin{array}{ll} u(t) & =\sum_{k=0}^{m-1}\frac{{\left(t-\phi \right)}^{\left(k\right)}}{k!}\Delta^{k}u\left(\phi \right)+\\ & \;\frac{1}{\Gamma (v)}\sum_{s=\phi +m-v}^{t-v}\left({\left(t-\sigma \left(s\right)\right)}^{\left(v-1\right)}\right)f\left(s+v-1,u\left(s+v-1\right)\right), \end{array}$$

*where the element $u_{0}\left(t\right)=\sum_{k=0}^{m-1}\frac{{\left(t-\phi \right)}^{\left(k\right)}}{k!}\Delta^{k}u\left(\phi \right)$ corresponds to initial iteration.*

**Remark** **1.** *Assuming the initial point is ϕ=0 and taking 0<v≤1, (6) is modified to*

(7) $$u\left(t\right)=u_{0}\left(t\right)+\frac{1}{\Gamma (v)}\sum_{s=1-v}^{t-v}\left({\left(t-\sigma \left(s\right)\right)}^{\left(v-1\right)}\right)f\left(s+v-1,u\left(s+v-1\right)\right).$$

*By letting s+v=j for (s+v)∈N, and utilizing the expansion ${\left(t-\sigma \left(s\right)\right)}^{\left(v-1\right)}=\frac{\Gamma (t-s)}{\Gamma (t-s-v+1)}$, the numerical formula with a global memory effect can be explicitly expressed as*

(8) $$u\left(t\right)=u_{0}\left(t\right)+\frac{1}{\Gamma (v)}\sum_{j=1}^{t}\frac{\Gamma (t-j+v)}{\Gamma (t-j+1)}f\left(j-1,u\left(j-1\right)\right).$$

## 3. Fractional Order Network with Indirect Coupling

Using the Caputo-like delta difference operator presented in (4), we introduce the fractionalized version of a network with two simple periodic discrete systems bidirectionally coupled. In [16], the integer-order dynamical network consists of two nodes and indirect coupling in discrete periodic oscillators. The outstanding feature of this complex network is that the systems used in the nodes cannot produce chaotic or hyperchaotic dynamics on their own; instead, such dynamics can only be achieved through indirect or direct coupling between them.

The state equations for the integer-order dynamical network are presented below. Specifically, the first node is denoted as $N_{1}$, and its state equations are given by [16]

(9) $$\begin{array}{c} X\left(n+1\right)=F\left(X\left(n\right)\right)+\hat{C}U_{1}\left(n\right). \end{array}$$

The second node $N_{2}$ is described by

(10) $$\begin{array}{c} Y\left(n+1\right)=F\left(Y\left(n\right)\right)+\hat{C}U_{2}\left(n\right), \end{array}$$

where $X={\left(x_{1}\left(n\right),\ldots ,x_{n}\left(n\right)\right)}^{T}\in R^{n}$ and $Y={\left(y_{1}\left(n\right),\ldots ,y_{n}\left(n\right)\right)}^{T}\in R^{n}$ are the state vectors of the coupled systems (9) and (10), $\hat{C}\in R^{n\times n}$ is a suitably chosen matrix indicating the indirect coupling, and $U_{1}\left(n\right)\in R^{n},$$U_{2}\left(n\right)\in R^{n}$ are the input signals of the systems in the nodes. The indirect coupling, introduced in [9], is reached by employing an intermediary system between the nodes to send information among the involved systems in the network; the indirect coupling is denoted as

(11) $$\begin{array}{c} h_{a}\left(n+1\right)=G\left(h_{a}\left(n\right)\right)-c\Phi \left(\eta X_{a}\left(n\right)+X_{b}\left(n\right)\right),\\ h_{b}\left(n+1\right)=G\left(h_{b}\left(n\right)\right)-c\Phi \left(\eta X_{b}\left(n\right)+X_{a}\left(n\right)\right), \end{array}$$

where $h_{a}\left(n\right)={\left[h_{a1}\left(n\right),h_{a2}\left(n\right)\right]}^{T}$ and $h_{b}\left(n\right)={\left[h_{b1}\left(n\right),h_{b2}\left(n\right)\right]}^{T}$ are the state vectors of the intermediate systems (11), and $G=[-1,1;-\zeta_{1},-\zeta_{2}]$ with $\zeta_{1}$, $\zeta_{2}\in R^{+}$; meanwhile, $\Phi \in R^{n\times n}$ is an appropriately chosen matrix, which couples the variables of the systems (9) and (10) within the intermediary systems (11), and c≠0 is the coupling strength, whereas η is a decompensation (bifurcation) parameter that allows us to build the route to chaos and hyperchaos for the coupled oscillators (9) and (10). To enable indirect coupling of the systems, we introduce the following input signals:

(12) $$\begin{array}{c} U_{1}\left(n\right)=h_{a}\left(n\right),\\ U_{2}\left(n\right)=h_{b}\left(n\right). \end{array}$$

**Remark** **2.** *The communication, or interaction, between the systems (9) and (10) is indirect and promoted through (12).*

In order to address the interesting problem of the emergence of hyperchaos through the coupling of naturally non-chaotic systems (see refs. [16,26]), we use the state equations of two simple autonomous discrete systems employed to generate complex dynamics as follows:

(13) $$\begin{array}{c} X\left(n+1\right)=\left[\begin{array}{c} \sin(x_{2}\left(n\right))\\ bx_{2}\left(n\right) \end{array}\right]+\hat{C}U_{1}\left(n\right), \end{array}$$

(14) $$\begin{array}{c} Y\left(n+1\right)=\left[\begin{array}{c} \sin(y_{2}\left(n\right))\\ by_{2}\left(n\right) \end{array}\right]+\hat{C}U_{2}\left(n\right). \end{array}$$

**Figure 1:**
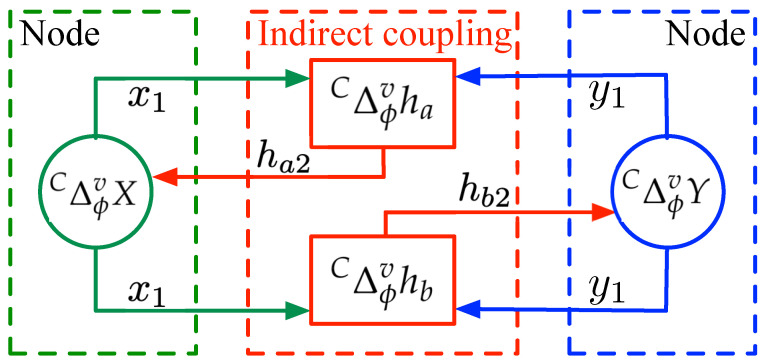
Representation of the proposed network with indirect coupling.

Employing the integer-order difference Equation (1) and the Caputo difference operator of Definition 2 in the nodes (9) and (10) and in the intermediary system (11), respectively, we obtain the fractional order dynamical network with two nodes and indirect coupling in discrete periodic oscillators as follows:

(15) $$\begin{array}{cc} {}^{C}\Delta_{\phi }^{v}X\left(t\right) & =F\left(t+v-1\right)+\hat{C}U_{1}\left(t+v-1\right)-X\left(t+v-1\right), \end{array}$$

(16) $$\begin{array}{cc} {}^{C}\Delta_{\phi }^{v}Y\left(t\right) & =F\left(t+v-1\right)+\hat{C}U_{2}\left(t+v-1\right)-Y\left(t+v-1\right), \end{array}$$

(17) $$\begin{array}{c} {}^{C}\Delta_{\phi }^{v}h_{a}\left(t\right)=G\left(h_{a}\left(t+v-1\right)\right)-c\Phi \left(\eta X_{a}\left(t+v-1\right)+X_{b}\left(t+v-1\right)\right)-h_{a}\left(t+v-1\right),\\ {}^{C}\Delta_{\phi }^{v}h_{b}\left(t\right)=G\left(h_{b}\left(t+v-1\right)\right)-c\Phi \left(\eta X_{b}\left(t+v-1\right)+X_{a}\left(t+v-1\right)\right)-h_{b}\left(t+v-1\right), \end{array}$$

where 0<v≤1 is the fractional order, $t\in N_{\phi +1-v}$, with ϕ defining the starting point. In Figure 1, the graphical representation of the network with indirect coupling is depicted.

Using the delta fractional difference presented in Theorem 1, we can represent the equivalent discrete integrals for 0<v≤1 as

(18) $$\begin{array}{ll} X(t) & =X\left(\phi \right)+\frac{1}{\Gamma (v)}\sum_{s=\phi +1-v}^{t-v}\left({\left(t-\sigma \left(s\right)\right)}^{\left(v-1\right)}\right)(F\left(s+v-1\right)+\\ & \;\hat{C}U_{1}\left(s+v-1\right)-X\left(s+v-1\right)), \end{array}$$

(19) $$\begin{array}{ll} Y(t) & =Y\left(\phi \right)+\frac{1}{\Gamma (v)}\sum_{s=\phi +1-v}^{t-v}\left({\left(t-\sigma \left(s\right)\right)}^{\left(v-1\right)}\right)(F\left(s+v-1\right)+\\ & \;\hat{C}U_{2}\left(s+v-1\right)-Y\left(s+v-1\right)), \end{array}$$

and

(20) $$\begin{array}{cc} h_{a}\left(t\right) & =h_{a}\left(\phi \right)+\frac{1}{\Gamma (v)}\sum_{s=\phi +1-v}^{t-v}\left({\left(t-\sigma \left(s\right)\right)}^{\left(v-1\right)}\right)(G\left(h_{a}\left(s+v-1\right)\right)-\\ \; & \;c\Phi \left(\eta X_{a}\left(s+v-1\right)+X_{b}\left(s+v-1\right)\right)-h_{a}\left(t+v-1\right)), \end{array}$$

(21) $$\begin{array}{cc} h_{b}\left(t\right) & =h_{b}\left(\phi \right)+\frac{1}{\Gamma (v)}\sum_{s=\phi +1-v}^{t-v}\left({\left(t-\sigma \left(s\right)\right)}^{\left(v-1\right)}\right)(G\left(h_{a}\left(s+v-1\right)\right)-\\ \; & \;c\Phi \left(\eta X_{b}\left(s+v-1\right)+X_{a}\left(s+v-1\right)\right)-h_{b}\left(t+v-1\right)), \end{array}$$

where (s+v)∈N, letting s+v=j and considering the discrete kernel function as

(22) $$\frac{1}{\Gamma (v)}\left({\left(t-\sigma \left(s\right)\right)}^{\left(v-1\right)}\right)=\frac{\Gamma (t-s)}{\Gamma (v)\Gamma (t-s-v+1)}.$$

The numerical Equations (18)–(21) can be explicitly presented. From this point forward, we assume that the starting point is ϕ=0 and the fractional order is 0<v≤1. The iterative process for the numerical Equations (18)–(21) can be expressed as follows

(23) $$\begin{array}{cc} X(n)= & X\left(0\right)+\frac{1}{\Gamma (v)}\sum_{j=1}^{n}\frac{\Gamma (n-j+v)}{\Gamma (n-j+1)}\left(F\left(j-1\right)+\hat{C}U_{1}\left(j-1\right)-X\left(j-1\right)\right), \end{array}$$

(24) $$\begin{array}{cc} Y(n)= & Y\left(0\right)+\frac{1}{\Gamma (v)}\sum_{j=1}^{n}\frac{\Gamma (n-j+v)}{\Gamma (n-j+1)}\left(F\left(j-1\right)+\hat{C}U_{2}\left(j-1\right)-Y\left(j-1\right)\right), \end{array}$$

(25) $$\begin{array}{ll} h_{a}\left(n\right)= & h_{a}\left(0\right)+\frac{1}{\Gamma (v)}\sum_{j=1}^{n}\frac{\Gamma (n-j+v)}{\Gamma (n-j+1)}(G\left(h_{a}\left(j-1\right)\right)-c\Phi (\eta X_{a}\left(j-1\right)+\\ & \;\;X_{b}\left(j-1\right))-h_{a}\left(j-1\right)), \end{array}$$

(26) $$\begin{array}{ll} h_{b}\left(n\right)= & h_{a}\left(0\right)+\frac{1}{\Gamma (v)}\sum_{j=1}^{n}\frac{\Gamma (n-j+v)}{\Gamma (n-j+1)}(G\left(h_{b}\left(j-1\right)\right)-c\Phi (\eta X_{b}\left(j-1\right)+\\ & \;\;X_{a}\left(j-1\right))-h_{b}\left(j-1\right)). \end{array}$$

When the order of Equations (23)–(26) is v=1, it is reduced to the classical integer-order difference network with indirect coupling given in (9)–(11), respectively.

**Remark** **3.** *The fractionalized Equations (18)–(21) and (23)–(26) differ from integer-order equations in that they possess a discrete kernel function, resulting in the states being dependent on previous information to determine the present state. This phenomenon is known as the memory effect.*

## 4. Dynamic Analysis of the Fractional Order Network with Indirect Coupling

To gain a deeper understanding of the behavior of the discrete fractional order network with indirect coupling given in (15)–(17), we perform a dynamic analysis of the network by studying the behavior of the network as a function of its parameters, focusing on phase portraits, temporal series, and bifurcation diagrams. We then analyze the maximal Lyapunov exponent of the network to gain insight into its underlying dynamics.

**Figure 2:**
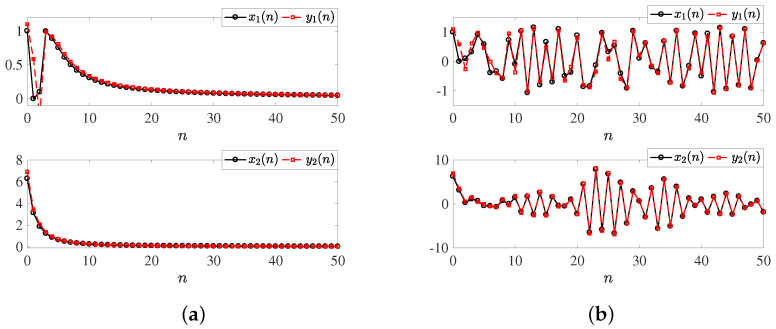
Temporal dynamics of the states $x_{1}\left(n\right)$, $x_{2}\left(n\right)$, $y_{1}\left(n\right)$, and $y_{2}\left(n\right)$, with initial condition sets X(0), Y(0) and fractional order v=0.9, (**a**) with coupling $\hat{C}=\left[0,\;0;\;0,\;0\right]$, (**b**) with coupling $\hat{C}=\left[0,\;0;\;0,\;1\right]$ and Φ=[0,0;1,0], respectively.

The nodes are uncoupled when the matrix $\hat{C}=\left[0,\;0;\;0,\;0\right]$; as a consequence, there exists no complex behavior, as shown in the temporal series depicted in Figure 2a, considering the parameter value b=0.5, initial conditions set as $X\left(0\right)={\left[1,\;2\pi \right]}^{T}$ and $Y\left(0\right)={\left[1.1,\;2.2\pi \right]}^{T}$, and v=0.9 being the fractional order. Meanwhile, in Figure 2b, the coupling interactions are enabled considering the first state variable to couple the autonomous fractional discrete-order systems (15) and (16) using $\hat{C}=\left[0,\;0;\;0,\;1\right]$, Φ=[0,0;1,0], c=1, $\zeta_{1}=0.1$, $\zeta_{2}=0.6$, η=0.5, v=0.9, and X(0), Y(0) sets, respectively, with the goal of having a more comprehensive understanding of the emerging dynamics caused by the interaction of the fractional order systems.

**Figure 3:**
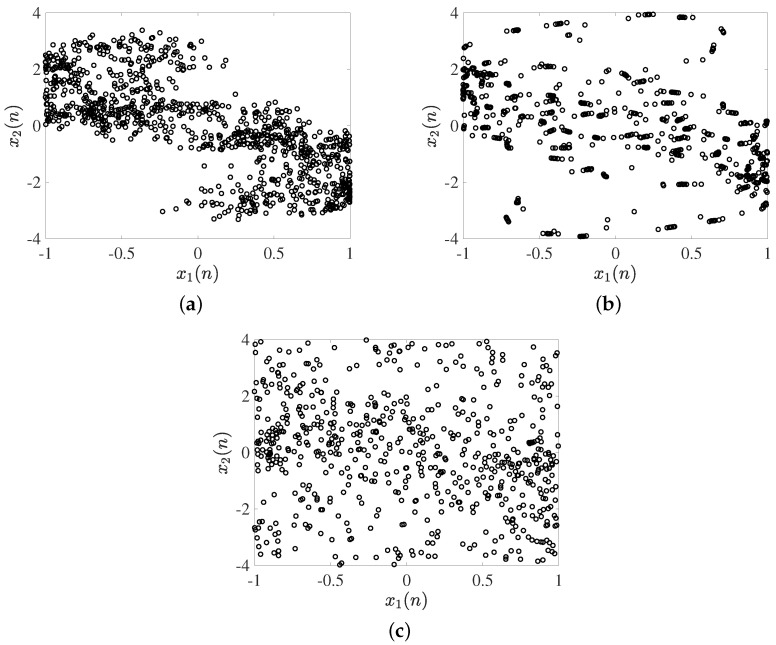
Phase portraits of fractionalized system (15) with different fractional orders: (**a**) fractional order v=1, (**b**) fractional order v=0.99, (**c**) fractional order v=0.9.

Figure 3 shows the phase portrait of fractionalized system (15), considering the numerical formula expressed in (23)–(26) by choosing the initial conditions as X(0) and Y(0) for three different scenarios. Figure 3a considers a fractional order v=1, Figure 3b a fractional order v=0.99, and Figure 3c a fractional order v=0.9. Considering n=1000 iterations, the phase plane was performed, discarding the first 100 values.

### Bifurcation Diagrams and Maximal Lyapunov Exponent

**Figure 4:**
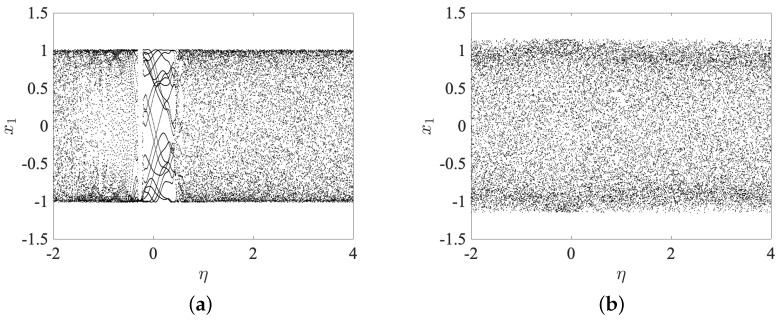
Bifurcation diagrams where η acts as a critical parameter with initial conditions sets X(0), Y(0). (**a**) For v=0.99, (**b**) for v=0.9, respectively.

A bifurcation diagram is a graphical representation of the qualitative behavior of a dynamic system as one or more of its parameters are varied [38]. It is a valuable tool for understanding the types of behavior exhibited by the system and the conditions under which such behavior occurs. In the case of a discrete fractional order chaotic system, the bifurcation diagram can reveal a wide range of complex dynamics, including periodic and chaotic behavior. To generate the bifurcation diagram, we vary the bifurcation parameter while keeping all other parameters constant and observing the resulting behavior of the system. The range of the bifurcation parameter and the step size used in the analysis can significantly affect the features observed in the diagram [39]. In the first instance, the bifurcation parameter η is selected as a critical parameter, and a step size of Δη=0.001 is used. The fractional order is set to v=0.99, and the bifurcation parameter is varied over the interval η∈[−2,4]. The resulting bifurcation diagram, shown in Figure 4a, reveals two regions where complex behavior arises, given by the intervals [−2,−0.37]∪[0.46,4]. Within these regions, the system exhibits periodic and chaotic behavior. In the second instance, a similar range is considered for the bifurcation parameter, and the fractional order is updated to v=0.9. In Figure 4b, the resulting bifurcation diagram shows that complex behavior always emerges for the interval selected. This indicates that the selection of the fractional order can have a significant impact on the dynamics of the system and should be carefully considered when analyzing such systems.

**Figure 5:**
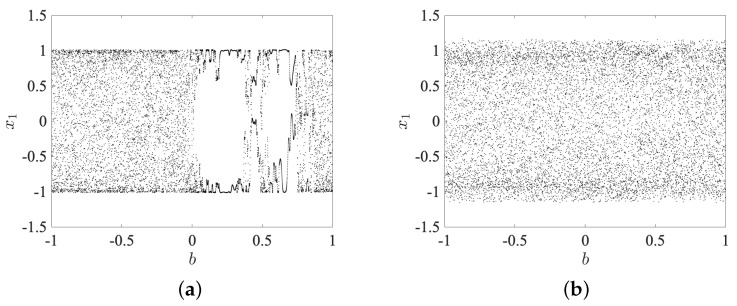
Bifurcation diagrams where *b* acts as a critical parameter with initial condition sets X(0), Y(0). (**a**) For v=0.99, (**b**) for v=0.9, respectively.

Moreover, in the second stage, the bifurcation diagrams where the parameter *b* acts as a critical parameter are shown in Figure 5. The parameter *b* is varied according to step size Δd=0.001. Figure 5a shows the bifurcation diagram when the fractional order v=0.99 and varying the bifurcation parameter in an interval b∈[−1,1]. It is observed that there are two regions where complex behavior emerges, given as [−1,−0.09]∪[0.7,1]. In Figure 5b, a similar range is considered, and now the fractional order was updated to v=0.9. It shows that complex behavior always emerges for the interval selected. The fractional order determines the memory and non-locality in the system, which can significantly impact the behavior of the system. In particular, modifying the fractional order can change the stability of the system, the type of bifurcations that occur, and the range of complex dynamics that are observed in the bifurcation diagram.

**Figure 6:**
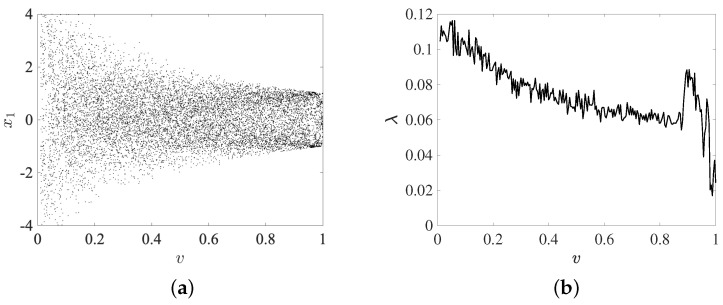
Bifurcation diagram and maximal Lyapunov exponent of the state $x_{1}$ by varying the parameter *v* in the interval v∈[0.01,1] with initial condition sets X(0), Y(0). (**a**) Bifurcation diagrams, (**b**) maximal Lyapunov exponent.

In addition, in order to explore the presence of chaos, we consider the bifurcation diagram and maximum Lyapunov exponent of the state $x_{1}$ by varying the parameter *v* in the interval v∈[0.01,1]. In Figure 6a, the bifurcation diagram is obtained by considering the fractional order *v* as a critical parameter. It is varied according to step Δv=0.001 and using $\hat{C}=\left[0,\;0;\;0,\;1\right]$, Φ=[0,0;1,0], c=1, $\zeta_{1}=0.1$, $\zeta_{2}=0.6$, η=0.5, v=0.9, and X(0), Y(0) sets, respectively. It shows that complex behavior always emerges for the interval selected. We also compute the maximal Lyapunov exponent of the system using the Jacobian matrix algorithm [40,41,42] shown in Figure 6b, which is fundamental in the analysis of dynamical systems as it provides a way to measure the rates at which nearby trajectories either converge or diverge in phase space [43]. The maximal Lyapunov exponent is a quantitative measure of the sensitivity in the system to initial conditions and is a valuable indicator of chaos [44]. It also coincides with Floquet multipliers for periodic orbits, which can help determine the significance of linearly unstable periodic orbits within a chaotic attractor. In this way, the positive multipliers of such orbits can serve as a measure of their relative importance to the overall chaotic dynamics [45]. As can be observed in the bifurcation and the maximal Lyapunov exponent represented in Figure 6 upon varying the parameter *v*, the systems (15)–(17) exhibits complex dynamics. In particular, the systems (15)–(17) is chaotic when the parameter v∈[0.01,1], where the value of the maximal Lyapunov exponents is positive.

## 5. Complexity Analysis via Spectral Entropy

Spectral entropy (SE) provides a quantitative characterization of the randomness and diversity of dynamics of the system, which makes it a valuable tool for analyzing complex behavior. It is based on the spectral properties of the time series of the system [46,47]. For instance, in Ref. [48], SE can identify global topology variations better than traditional probability distribution entropy, capturing the overall structure of the network rather than just local features. It quantifies the disorder or randomness of the energy distribution across the different frequencies in the time series being calculated through a Fourier transform. Additionally, SE is based on information diffusion, which may make it more effective in capturing dynamic changes in the network over time.

The spectral entropy of a signal is defined according to

(27) $$H=-\frac{1}{\log_{2}N}\sum_{k}P\left(k\right)\log_{2}P(k),$$

where P(k) is the normalized power spectrum at the frequency bin k∈[0,1,…,N−1] and *N* is the number of frequency bins in the power spectrum. The spectral entropy ranges between 0 and 1. A lower spectral entropy value indicates a more ordered and predictable signal, while a higher value indicates a more complex and unpredictable signal.

In the context of this paper, spectral entropy is used to measure the emergence of complex behavior when the indirect coupling between fractional order discrete nodes is activated by analyzing the power spectrum of the chaotic series generated. Moreover, spectral entropy is used to track changes in the complexity of the network over time and to identify critical transitions or bifurcations in the dynamics of the nodes.

**Figure 7:**
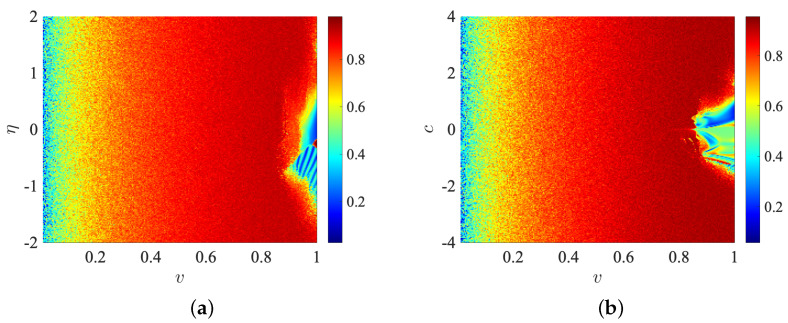
Spectral entropy *b* acts as a critical parameter with initial conditions sets X(0), Y(0). (**a**) for v=0.99 (**b**) for v=0.9, respectively., respectively.

Figure 7 shows the spectral entropy of the network (15) and (16) with indirect coupling (17), when the fractional order *v* and the parameters η and *c* are varied. Specifically, Figure 7a exhibits the SE plane of fractional order *v* versus the parameters η in the intervals v∈[0.01,1] and η∈[−2,2], respectively. Here, we established the parameters as c=0.5, $\zeta_{1}=0.1$, $\zeta_{2}=0.6$, b=0.5, and initial conditions sets X(0) and Y(0). Figure 7b shows the SE plane of fractional order *v* versus *c* parameter (coupling strength). It is obtained by defining the parameters as in the previous case, with the exception of η, defined as η=0.5 and varying the fractional order in the interval v∈[0.01,1] and the parameter c∈[−4,4], respectively.

## 6. FPGA Implementation of Complex Network with Two Fractional Order Chaotic Nodes

**Figure 8:**
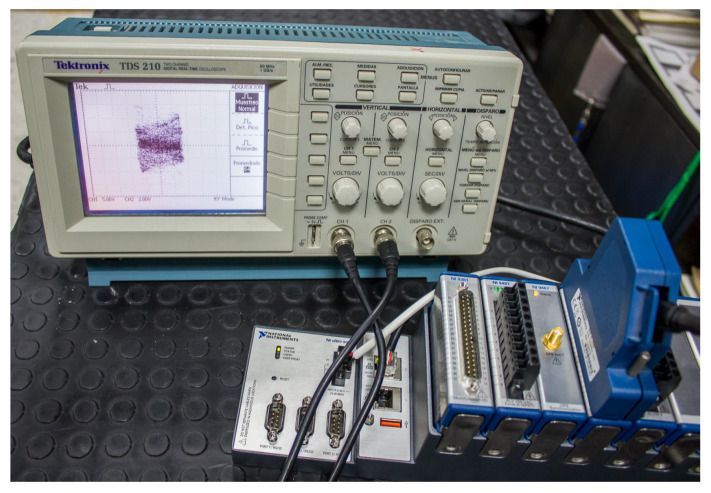
Setup of the Xilinx Zynq−7000 XC7Z020 FPGA implementation.

Field-programmable gate arrays (FPGAs) provide a flexible and efficient platform for implementing complex systems. This section proposes an FPGA implementation of the complex network shown in Section 3. Figure 8 shows the experimental implementation. We use the Xilinx Zynq−7000 XC7Z020 FPGA chip and the National Instruments LabVIEW FPGA compiler. To implement the network on an FPGA, we use single-precision floating-point arithmetic to compute the fractional order derivatives, the nonlinear functions of the nodes, and the indirect coupling terms. We consider the short-memory principle to reduce computational complexity, using a memory length L=10 samples.

**Figure 9:**
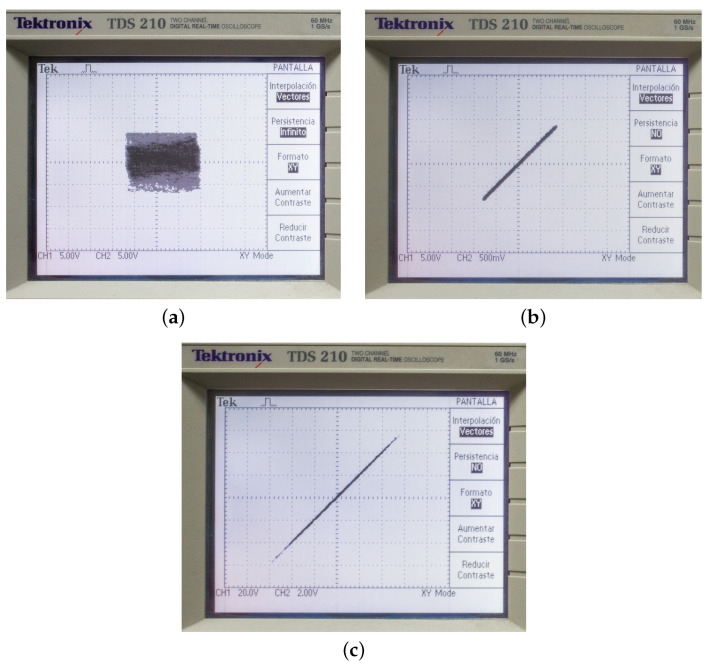
FPGA implementation results. (**a**) The phase plane $x_{1}$ versus $x_{2}$, (**b**) the phase plane $x_{1}$ versus $y_{1}$, (**c**) the phase plane $x_{2}$ versus $y_{2}$.

To validate the proposed FPGA implementation, we compare the implementation results achieved with those obtained from software simulations of the same system. We also analyze the resource usage and performance of the FPGA implementation. The resources used for the design are presented in Table 1. The clock frequency used was 40 MHz, and the minimum execution time achieved for the implemented system was 30 microseconds. The experimental results are shown in Figure 9. They were obtained from an oscilloscope Tektronix TDS 210 with a bandwidth of 60 MHz. In Figure 9a we present the phase plane $x_{1}$ versus $x_{2}$. Figure 9b shows the phase plane $x_{1}$ versus $y_{1}$. The phase plane $x_{2}$ versus $y_{2}$ is depicted in Figure 9c.

## 7. Conclusions

In this study, we investigated the emergence of chaos in complex dynamical networks with indirect coupling and discrete systems, both utilizing fractional order. We utilized indirect coupling to produce complex dynamics in the network. Moreover, the analysis of the fractionalized version of the complex network and indirect coupling was carried out by temporal series, phase planes, bifurcation diagrams, and Lyapunov exponent. The selection of the fractional order in the studied system can profoundly impact its dynamics. The fractional order implies a non-locality and memory effect within the system, which can influence its behavior. In particular, changes to the fractional order can alter the stability of the system, the nature of its temporal series, phase planes, bifurcation diagrams, Lyapunov exponent, and the range of complex dynamics observed. We compute the spectral entropy of the chaotic series to quantify the complexity of the network. Our results show that the dynamic coupling of discrete fractional nodes can increase the complexity of the complex network, leading to higher entropy values and chaos. As a final step, we demonstrate the feasibility of implementing the complex network on a field-programmable gate array (FPGA), confirming its hardware realizability where, for example, the perspectives for generalizing this method for multi-node networks are high; although it is true that the processing required for the experimental implementation using only one FPGA may be demanding, solutions can be sought, such as using an FPGA network to implement each systems (13) and (14) within each of the FPGAs in the network. We consider that there are different directions for future research. For example, exploring the impact of different types of coupling and investigating the behavior of networks with a different number of nodes could lead to further insights and a deeper understanding of the behavior of complex dynamical networks, the control of emerging behavior, and their applications in secure communications.

## Figures and Tables

**Table 1:** FPGA chip resource usage for the Xilinx Zynq-7000 XC7Z020 FPGA chip.

Device Resources	Total	Used	Percent (%)
Total Slices	13,300	10,588	79.6
Slice Registers	106,400	26,778	25.2
Slices LUTs	53,200	34,283	64.4
Block RAMs	140	7	5
DSP48s	220	22	10

## References

[1] Agieva M., Korolev A., Ougolnitsky G. (2020). Modeling and simulation of impact and control in social networks with application to marketing. Mathematics.

[2] Zareie A., Sakellariou R. (2023). Centrality measures in fuzzy social networks. Inf. Syst..

[3] Guze S. (2019). Graph theory approach to the vulnerability of transportation networks. Algorithms.

[4] Müller F. (2023). Link and edge weight prediction in air transport networks—An RNN approach. Phys. A Stat. Mech. Its Appl..

[5] Zambrano-Serrano E., Munoz-Pacheco J.M., Anzo-Hernández A., Félix-Beltrán O.G., Guevara-Flores D.K. (2022). Synchronization of a cluster of *β*-cells based on a small-world network and its electronic experimental verification. Eur. Phys. J. Spec. Top..

[6] Cha J., Lee I. (2020). Single-cell network biology for resolving cellular heterogeneity in human diseases. Exp. Mol. Med..

[7] Milić M., Milojković J., Jeremić M. (2022). Optimal Neural Network Model for Short-Term Prediction of Confirmed Cases in the COVID-19 Pandemic. Mathematics.

[8] Arellano-Delgado A., López-Gutiérrez R., Méndez-Ramírez R., Cardoza-Avendaño L., Cruz-Hernández C. (2021). Dynamic coupling in small-world outer synchronization of chaotic networks. Phys. D Nonlinear Phenom..

[9] Ramirez J.P., Arellano-Delgado A., Nijmeijer H. (2018). Enhancing master-slave synchronization: The effect of using a dynamic coupling. Phys. Rev. E.

[10] de Jonge W., Ramirez J.P., Nijmeijer H. (2019). Dynamic coupling enhances network synchronization. IFAC-PapersOnLine.

[11] Leonel Rocha J., Carvalho S. (2023). Complete dynamical networks: Synchronization, information transmission and topological order. Discontinuity Nonlinearity Complex..

[12] Rocha J.L., Carvalho S. (2021). Information theory, synchronization and topological order in complete dynamical networks of discontinuous maps. Math. Comput. Simul..

[13] Caneco A., Rocha J.L., Grácio C. (2009). Topological entropy in the synchronization of piecewise linear and monotone maps: Coupled duffing oscillators. Int. J. Bifurc. Chaos.

[14] Zambrano-Serrano E., Anzo-Hernández A. (2021). A novel antimonotic hyperjerk system: Analysis, synchronization and circuit design. Phys. D Nonlinear Phenom..

[15] Buscarino A., Fortuna L., Patanè L. (2019). Master-slave synchronization of hyperchaotic systems through a linear dynamic coupling. Phys. Rev. E.

[16] Arellano-Delgado A., Méndez-Ramírez R.D., López-Gutiérrez R.M., Murillo-Escobar M.A., Cruz-Hernández C. (2023). Enhancing the emergence of hyperchaos using an indirect coupling and its verification based on digital implementation. Nonlinear Dyn..

[17] Zambrano-Serrano E., Posadas-Castillo C., Platas-Garza M. (2022). Coexistence of Chaotic Attractors in a Four-Dimensional Memristor-Based Chaotic Nonlocal System: Analysis and FPGA Implementation. Applications of Fractional Calculus to Modeling in Dynamics and Chaos.

[18] Yao X., Liu Y., Zhang Z., Wan W. (2021). Synchronization rather than finite-time synchronization results of fractional-order multi-weighted complex networks. IEEE Trans. Neural Netw. Learn. Syst..

[19] Clemente-López D., Munoz-Pacheco J.M., Rangel-Magdaleno J.D.J. (2023). A Review of the Digital Implementation of Continuous-Time Fractional-Order Chaotic Systems Using FPGAs and Embedded Hardware. Arch. Comput. Methods Eng..

[20] Zambrano-Serrano E., Munoz-Pacheco J.M., Serrano F.E., Sánchez-Gaspariano L.A., Volos C. (2021). Experimental verification of the multi-scroll chaotic attractors synchronization in PWL arbitrary-order systems using direct coupling and passivity-based control. Integration.

[21] Singh P.P., Borah M., Datta A., Jafari S., Roy B.K. (2023). Integer cum fractional ordered active-adaptive synchronization to control vasospasm in chaotic blood vessels to reduce risk of COVID-19 infections. Int. J. Comput. Math..

[22] Yan B., Parastesh F., He S., Rajagopal K., Jafari S., Perc M. (2022). Interlayer and intralayer synchronization in multiplex fractional-order neuronal networks. Fractals.

[23] Echenausía-Monroy J.L., Rodríguez-Martíne C., Ontañón-García L., Alvarez J., Pena Ramirez J. (2021). Synchronization in dynamically coupled fractional-order chaotic systems: Studying the effects of fractional derivatives. Complexity.

[24] García-Grimaldo C., Bermudez-Marquez C.F., Tlelo-Cuautle E., Campos-Cantón E. (2023). FPGA Implementation of a Chaotic Map with No Fixed Point. Electronics.

[25] Cassal-Quiroga B., Gilardi-Velázquez H., Campos-Cantón E. (2022). Multistability Analysis of a Piecewise Map via Bifurcations. Int. J. Bifurc. Chaos.

[26] Arellano-Delgado A., López-Gutiérrez R.M., Murillo-Escobar M.A., Cardoza-Avendaño L., Cruz-Hernández C. (2017). The emergence of hyperchaos and synchronization in networks with discrete periodic oscillators. Entropy.

[27] Miller K.S., Ross B. Fractional difference calculus. Proceedings of the International Symposium on Univalent Functions, Fractional Calculus and Their Applications.

[28] Trujillo J.J., Ungureanu V.M. (2018). Optimal control of discrete-time linear fractional-order systems with multiplicative noise. Int. J. Control.

[29] Djennoune S., Bettayeb M., Al-Saggaf U.M. (2019). Synchronization of fractional-order discrete-time chaotic systems by an exact delayed state reconstructor: Application to secure communication. Int. J. Appl. Math. Comput. Sci..

[30] Andrei L., Caus V.A. (2021). A Generalized Class of Functions Defined by the q-Difference Operator. Symmetry.

[31] Saadeh R., Abbes A., Al-Husban A., Ouannas A., Grassi G. (2023). The Fractional Discrete Predator–Prey Model: Chaos, Control and Synchronization. Fractal Fract..

[32] Al-Shbeil I., Djenina N., Jaradat A., Al-Husban A., Ouannas A., Grassi G. (2023). A New COVID-19 Pandemic Model Including the Compartment of Vaccinated Individuals: Global Stability of the Disease-Free Fixed Point. Mathematics.

[33] Ma W., Li Z., Ma N. (2022). Synchronization of discrete fractional-order complex networks with and without unknown topology. Chaos Interdiscip. J. Nonlinear Sci..

[34] Zambrano-Serrano E., Bekiros S., Platas-Garza M.A., Posadas-Castillo C., Agarwal P., Jahanshahi H., Aly A.A. (2021). On chaos and projective synchronization of a fractional difference map with no equilibria using a fuzzy-based state feedback control. Phys. A Stat. Mech. Its Appl..

[35] Atici F., Eloe P. (2009). Initial value problems in discrete fractional calculus. Proc. Am. Math. Soc..

[36] Abdeljawad T. (2011). On Riemann and Caputo fractional differences. Comput. Math. Appl..

[37] Fulai C., Xiannan L., Yong Z. (2011). Existence Results for Nonlinear Fractional Difference Equation. J. Adv. Differ. Equ..

[38] Wiggins S., Wiggins S., Golubitsky M. (2003). Introduction to Applied Nonlinear Dynamical Systems and Chaos.

[39] Drzewiecki G. (2021). Fundamentals of Chaos and Fractals for Cardiology.

[40] Wu G.C., Baleanu D. (2015). Jacobian matrix algorithm for Lyapunov exponents of the discrete fractional maps. Commun. Nonlinear Sci. Numer. Simul..

[41] Danca M.F. (2022). Fractional order logistic map: Numerical approach. Chaos Solitons Fractals.

[42] Abbes A., Ouannas A., Shawagfeh N., Jahanshahi H. (2023). The fractional-order discrete COVID-19 pandemic model: Stability and chaos. Nonlinear Dyn..

[43] Pikovsky A., Politi A. (2016). Lyapunov Exponents: A Tool to Explore Complex Dynamics.

[44] Li H., Shen Y., Han Y., Dong J., Li J. (2023). Determining Lyapunov exponents of fractional-order systems: A general method based on memory principle. Chaos Solitons Fractals.

[45] Oeri H., Goluskin D. (2022). Convex computation of maximal Lyapunov exponents. arXiv.

[46] Xiong P.Y., Jahanshahi H., Alcaraz R., Chu Y.M., Gómez-Aguilar J., Alsaadi F.E. (2021). Spectral entropy analysis and synchronization of a multi-stable fractional-order chaotic system using a novel neural network-based chattering-free sliding mode technique. Chaos Solitons Fractals.

[47] Asgharzadeh-Bonab A., Amirani M.C., Mehri A. (2020). Spectral entropy and deep convolutional neural network for ECG beat classification. Biocybern. Biomed. Eng..

[48] Su H., Chen D., Pan G.J., Zeng Z. (2021). Identification of network topology variations based on spectral entropy. IEEE Trans. Cybern..

